# Insights into Amyotrophic Lateral Sclerosis from a Machine Learning Perspective

**DOI:** 10.3390/jcm8101578

**Published:** 2019-10-01

**Authors:** Jonathan Gordon, Boaz Lerner

**Affiliations:** Industrial Engineering & Management Department, Ben-Gurion University of the Negev, Beer-Sheva 84105, Israel

**Keywords:** ALS, ALS functional rating scale (ALSFRS), Bayesian networks, disease progression, disease state, feature selection, machine learning, ordinal classification, PRO-ACT database

## Abstract

**Objective:** Amyotrophic lateral sclerosis (ALS) disease state prediction usually assumes linear progression and uses a classifier evaluated by its accuracy. Since disease progression is not linear, and the accuracy measurement cannot tell large from small prediction errors, we dispense with the linearity assumption and apply ordinal classification that accounts for error severity. In addition, we identify the most influential variables in predicting and explaining the disease. Furthermore, in contrast to conventional modeling of the patient’s total functionality, we also model separate patient functionalities (e.g., in walking or speaking). **Methods:** Using data from 3772 patients from the Pooled Resource Open-Access ALS Clinical Trials (PRO-ACT) database, we introduce and train ordinal classifiers to predict patients’ disease state in their last clinic visit, while accounting differently for different error severities. We use feature-selection methods and the classifiers themselves to determine the most influential variables in predicting the disease from demographic, clinical, and laboratory data collected in either the first, last, or both clinic visits, and the Bayesian network classifier to identify interrelations among these variables and their relations with the disease state. We apply these methods to model each of the patient functionalities. **Results:** We show the error distribution in ALS state prediction and demonstrate that ordinal classifiers outperform classifiers that do not account for error severity. We identify clinical and lab test variables influential to prediction of different ALS functionalities and their interrelations, and specific value combinations of these variables that occur more frequently in patients with severe deterioration than in patients with mild deterioration and vice versa. **Conclusions:** Ordinal classification of ALS state is superior to conventional classification. Identification of influential ALS variables and their interrelations help explain disease mechanism. Modeling of patient functionalities separately allows relation of variables and their connections to different aspects of the disease as may be expressed in different body segments.

## 1. Introduction

Despite medical and clinical progress since its discovery 150 years ago, the inner workings and mechanisms of amyotrophic lateral sclerosis (ALS) remain largely unknown [1,2,3]. However, it is understood that extending life expectancy and improving the quality of life of those afflicted depend on our understanding of the disease pathogenesis [4,5].

What has complicated clinical research and trials regarding ALS is the heterogeneity of the ALS population [2,4,6], which is characterized by variability among the patients in the disease progression rate, site of symptoms at onset, and survivability. Furthermore, it often becomes difficult to assess the influence of a certain therapy upon a group of patients due to the inherent heterogeneity among them [1,3,4]. It is clear today that a reliable individualized prediction of a patient’s disease progression rate could improve the ability to assess treatment influence in a clinical trial and reduce the number of patients necessary to achieve statistically significant results.

Machine learning (ML) has proven highly beneficial in the past when used for medical diagnosis and prediction from data. Lavrac [7] extensively describes the potential value of new and useful knowledge that can be derived by applying ML to large collections of medical data. Kononenko [8] provides an overview of intelligent analysis in medicine from an ML perspective, and further discusses future trends and the importance of ML in the medical research community, demonstrating that performance of algorithms such as semi-naive Bayes and neural network classifiers can improve upon physicians’ reliability of diagnosis with regard to ishaemic heart disease. Others [9] compare ML algorithms that predict mortality among patients with pneumonia and show that low predictive error rates can be achieved. The ability to predict mortality in pneumonia patients is useful to clinicians in making decisions regarding the optimal location for patient treatment. Lerner et al., [10,11,12] discuss how training the linear classifier, naive Bayesian classifier, multi-perceptron neural network, Bayesian neural network, and support vector machine based on well-discriminating features can achieve accurate classification of fluorescence in situ hybridization signals, which can in turn lead to useful detection of genetic abnormalities. Also, in a recent special issue of the *Journal of Clinical Medicine*, “The Future of Artificial Intelligence in Clinical Medicine”, close to 25 (!!) papers were published just on the use of ML in medicine (see, e.g., [13,14,15,16]).

In this study, we used ML algorithms and the Pooled Resources Open-Access Clinical Trials (PRO-ACT) database [17], which is the largest ALS clinical trials database ever created (Section 3.1), to build models that can effectively predict and analyze the ALS disease state as represented by the ALS functional rating scale (ALSFRS) [18]. ALSFRS is an accepted rating system used to monitor and measure patients’ disease state. It comprises ten items, each representing another patient functionality (e.g., in walking, swallowing, and breathing). Each ALSFRS item holds a value between 0 and 4 (0 representing complete inability with regard to the function, and 4 representing normal function), and the collection of these item values represents the patient’s disease state at a given time, and thus is a true descriptor for this state in designing prediction models. For the prediction, we employed physiological and lab test variables that were measured in the current and past clinic visits.

Our first contribution is that by not assuming any disease progression behavior, especially not that the deterioration of the ALSFRS follows a linear function [19], we mapped patient data collected along the disease course directly to ALSFRS values at a certain point in time.

While previous studies used conventional classifiers to predict ALS disease state and the classification accuracy to augment the classifier during learning and to evaluate its performance, it does not address the ordinal nature of ALSFRS, and thus does not consider different error severities differently. For example, predicting a value of ALSFRS is 2 or 1 when the real value is 3 accounts for different error severities, although considered similarly by the classification accuracy that only counts errors. Our second contribution is, therefore, the introduction of ordinal classification algorithms accounting differently, while being trained, for the different error severities, e.g., by penalizing a classifier predicting a patient’s walking functionality of 1, while it is 3, more than if it were predicting the functionality to be 2. We evaluated the ability of the ordinal classifiers in predicting the disease state for patients from the PRO-ACT database using the mean absolute error that accounts for the error severity and showed that ordinal classification is not only more informative than conventional classification, but also more accurate.

Our third contribution is the determination of the most influential variables on predicting the disease from among demographic, vital signs, and lab test variables recorded during the disease course using a feature-selection technique and ML classifiers. Besides simply identifying these important features, we trained Bayesian network classifiers that, beyond their prediction capability, expose interactions and demonstrate relationships between predictors and ALS patient functionality (measured by e.g., their walking, breathing, and speaking ALSFRS values), shedding some light on unknown mechanisms of the disease. In addition, after identifying functionalities that are highly correlated with each other, and grouping them into higher level representations of patient functions, we could map certain predictors to these high-level groupings. Then we found specific value combinations of important predictors that occur frequently in data of patients exhibiting severe deterioration with respect to certain functions in comparison to other value combinations of these predictors that were frequent in data of patients exhibiting mild deterioration in the same functions.

Our fourth contribution is that, while past research has viewed the state of the disease from a general perspective as represented by the sum of all ALSFRS items (i.e., the patients’ functionality is characterized by their ability in performing all daily tasks jointly), we examine the functional abilities separately, as if not necessarily originating from the same source, and train separate models for each of the different functions. A motivation for this separation comes from the fact that different functioning abilities deteriorate at different rates. For example, patients usually drop one point from normal in the Dressing and Hygiene sub-scores by 12 months, whereas Handwriting and Cutting Food sub-scores may take 12–24 months to drop by one point [20]. Based on these findings, we propose that rather than having one underlying mechanism for ALS, there should be several different mechanisms, each controlling a different aspect of the disease and being expressed in another body functionality.

Section 2 of this paper gives a short background of ALS. Section 3 describes our methodology, data, learning algorithms, and experiments conducted in this research. Section 4 contains an analysis of the experiment results, and Section 5 draws conclusions and discusses the research results.

## 2. Background to ALS

ALS is a devastating illness with an unknown pathogenesis. It is an idiopathic fatal neurodegenerative disease of the human motor system [4,5,21]. Despite medical and clinical progress in recent decades, the disease is still not visibly affected by the different therapies available today [1,3,6].

ALS attacks both the upper and lower motor neurons [22,23]. Its overall pathological hallmarks are the degeneration and loss of motor neurons with astrocytic gliosis [2,3]. Clinical features include loss of neurons at all levels of the motor system: from the cortex to the anterior horn of the spinal cord and motor nuclei in the brainstem [1,5]. In recent years, advancements in multi-modal neuroimaging have confirmed that ALS is a multi-system neurodegenerative disease involving, not only the motor system, but also the frontal cortex and other structures [4,23]. The recently developed Braak neuropathological staging system has shown that ALS disseminates in a sequential regional pattern from the precentral motor cortex, brainstem motor nuclei, and spinal cord motor neurons to prefrontal areas, postcentral cortex and basal ganglia, and lastly to the temporal cortex, including the hippocampus.

ALS is a rare disease; approximately 2 out of 100,000 people will be diagnosed with it every year [1,2]. ALS is mainly a sporadic disease (i.e., the cause(s) of the disease are unknown), but about 10% of ALS cases are familial. Familial ALS is more easily identified when there is a positive family history, but in the absence of such history, an early age of onset, atypical rapid or slow disease progression, pure lower motor neuron presentation, or the presence of dementia may alert to a familial etiology [24]. Although approximately 60% of the genes associated with familial ALS have been identified, the classification of sporadic and familial ALS is not clear because the same gene mutations may account for both types.

**Figure 1:**
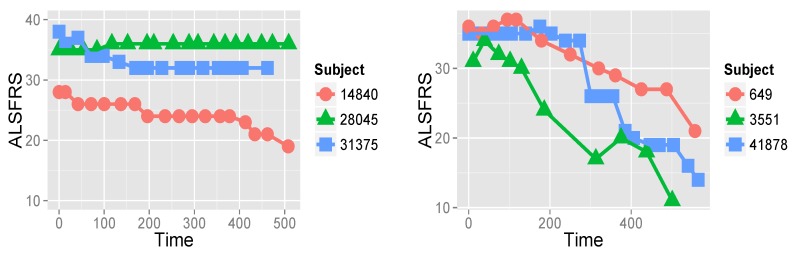
ALSFRS values measuring the patient’s ability in ten functions, such as walking, breathing, and dressing, of six patients from the PRO-ACT database over roughly 500 days. On the left, disease progression of three patients (subjects) is relatively moderate, whereas on the right, deterioration of three other patients is very fast.

Onset in ALS is normally after the age of 40 [3,5]. In around 75% of ALS cases, onset is in the limbs, and the remaining cases are bulbar-onset. The course of the disease is inexorably progressive, with 50% of patients dying within three years after onset, and about 20% of patients surviving between five and ten years. Among recorded symptoms are weakness (which may start in the hands or legs), slurred speech, dysphagia, dyspnea (shortness of breath), cognitive changes, sialorrhea (excess of saliva), depression and anxiety, and sleep disturbance [1,3,4]. The course of the appearance of these symptoms differs from patient to patient, as does the rate of disease progression. Some studies [25,26] show that the disease progression rate is heterogeneous, and that there may be sub-groups of patients that exhibit similar progression rates and patterns. To exemplify this, Figure 1 shows two groups of three representative patients that demonstrate different behaviors of disease progression. The first group represents moderate disease progression, whereas the second group is of patients for whom deterioration is very rapid.

This heterogeneity of the disease course, ranging from under a year to over ten years, is a substantial obstacle to understanding ALS and developing an effective treatment for it. Clinical trials held up to today have been largely unsuccessful in finding causes, solutions, or treatments [1,6,25], with only two approved medications that can only extend life expectancy for several months. The more heterogeneous a disease, the more difficult it is to predict how a given patient’s disease will progress, thus making it difficult to demonstrate the potential usefulness of a given therapy. A more accurate way to anticipate disease progression could therefore lead to meaningful improvements in clinical treatment and research and drug development [6,27,28]. Nevertheless, only a few studies have attempted to address the uncertainty veiling ALS using a data-driven approach.

Since its launch, the main goal of projects using the PROACT database has been the prediction of disease progression rate, primarily with the aim of reducing the size of clinical trials necessary to reach statistical certainty [19,27,28]. For example, Zach et al., [28] discuss the many past and potential future benefits of applying ML to the PRO-ACT database. Additionally, Kuffner et al., [27] and Gomeni et al., [19] demonstrate this potential with respect to ALS by constructing statistical and mathematical models of the disease. In 2012, Prize4Life initiated the ALS prediction challenge, which challenged researchers to use the PRO-ACT database to predict the rate of disease progression [27], which was assumed to be linear, with a mean decrease of about one point per month in the sum of ten ALSFRS items, each for a different patient functionality (e.g., speaking, walking, swallowing, etc.). A combination of algorithms produced in the challenge showed potential reduction in the number of patients needed for clinical trials by up to 20.4% [27]. In a recent prediction challenge organized by Prize4Life [29], competitors were required to first stratify the patient population into sub-groups, identify important predictors for each such group, and leverage the stratification to improve prediction of patients’ disease progression rate and survival using the PRO-ACT database (A model called ML-BGU, submitted by the authors, came in 6th place out of 30 models [30] qualified in the sub-challenge of progression rate prediction using the PRO-ACT database, and was the only model of the first six that followed the above challenge requirements).

## 3. Methodology

**Figure 2:**
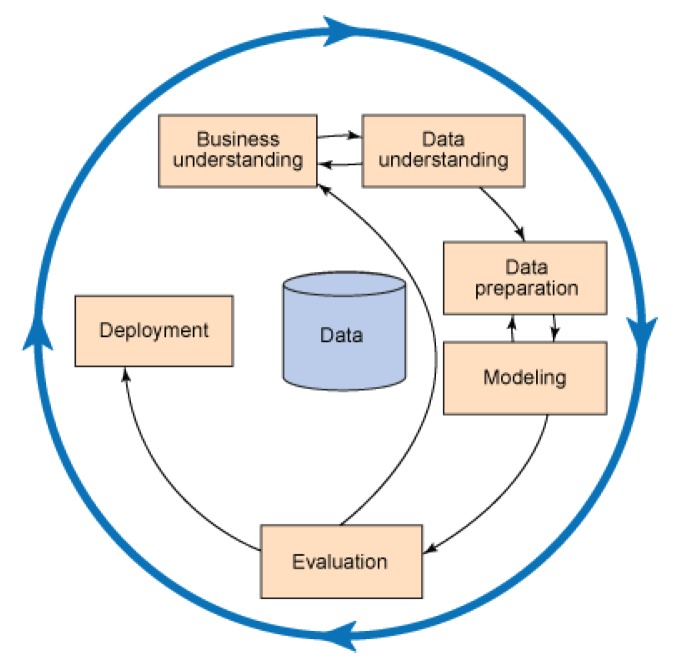
The basic schema of CRISP-DM.

The methodology of this research is based on the accepted CRISP-DM (Cross Industry Standard Process for Data Mining) methodology (Figure 2). The methodology represents an iterative work process that includes a business (which is medical in our case) understanding of the problem at hand, understanding the database, preparation of the data, modeling, evaluation of the model, and deployment [31]. Problem/business understanding has already been discussed in Section 2.

### 3.1. Data Understanding: The PRO-ACT Database

Data used in the preparation of this article were obtained from the PRO-ACT Database. In 2011, Prize4Life, in collaboration with the Northeast ALS Consortium, and with funding from the ALS Therapy Alliance, formed the PRO-ACT Consortium. The data available in the PRO-ACT Database has been volunteered by PRO-ACT Consortium members. The PRO-ACT database houses the largest ALS clinical trials dataset ever created, merging data from existing public and private clinical trials. The database contains over 8500 unique clinical patient records from 17 late-stage industry and academic clinical trials [17,28].

The database was received in the form of several different data tables. One of the challenges of working with the database is the heterogeneity of the tables, where each one may contain a different number of patients. A patient may or may not appear on a given table, and even on the same tables, different patients might have had different data collected about them. This is apparently due to the data collection process, which may vary greatly from one trial protocol to another. Table 1 summarizes the different data tables that comprise the database. In this study, we were especially interested in the data contained within the lab test tables because this type of data has not yet been studied thoroughly (and note that the PRO-ACT database does not include genetic data). We distinguished between three types of variables in the database:

- *Static variables*: Variables for which values were determined in one clinic visit and were no longer tested in later visits. These variables are fixed per patient and cannot change over time. Some examples are gender, ethnicity, time of onset, and diagnosis.
- *Temporal variables*: Variables whose values can change over time. They were tested at multiple clinic visits throughout the trials, and appear several times for each patient. Examples of these are pulse, blood pressure, weight, and laboratory test results.
- *Target variables*: The ALSFRS values for ten items. They too were measured in all clinic visits during the trial, and therefore are considered temporal data. We created the distinction between these and the regular temporal variables, as ALSFRS items have the role of target variables. These variables take values ranging from 0 to 4, 4 representing normal function, and 0 being the absolute loss of the respective function. Note that patients in the database have either ALSFRS or ALSFRS-R values documented. ALSFRS-R is a revised version of the ALSFRS system, which expands the respiratory function to three separate values [22]. For uniformity’s sake, we required that each patient have the same number of values for the respiratory function. Since ALSFRS cannot be expanded, it was necessary to “collapse” the ALSFRS-R respiratory values into one value (as in [32]). After consulting with experts at Prize4Life, it was decided that the value of Dyspnea would be the most accurate representation for respiratory capacity, for patients whose disease state was documented using the ALSFRS-R rating system.

### 3.2. Data Preparation

One of the biggest challenges that arose from working with the database was missing data. As mentioned above, the PRO-ACT database was collected by merging data from existing public and private clinical trials. This method of collection inevitably leads to inconsistencies in the data collected and missing values. Missing data were dealt with in several fashions.

As this study views the ALSFRS values as true depictors of the disease state in the database, and thus, as the target variable, records of all patients who did not have these values documented were rendered unusable for our purposes, a total of 4838 records of patients remained to be used in the training of the models.

Where it was possible to complete large portions of the missing data using external sources and medical consultation, data were completed. Such was the case for the FVC data table. FVC is an accepted medical test for determining respiratory capacity, which is shown to be a meaningful predictor of ALS survival [1,28]. For many patients, only the absolute value of the test was available, whereas the ratio between this value and the normal value in the overall population is the more meaningful predictor, yet was largely missing from the database (68.59% missing). By converting the absolute value to the percent of the normal value (using a normal values table supplied by Prize4Life), we were able to complete a large portion of the missing data. (After completion, only 0.43% of the values were missing).

For many of the variables in the database, missing values were so common that we decided that completion of the data would be impossible, so those variables were not used. Examples of these are variables regarding patient ethnicity and lab tests that were sparsely administered (e.g., fibrinogen, lactate dehydrogenase, and prothrombin (clotting time)). In cases in which a small percentage of variables known to be important were missing for groups of patients, we decided to keep the variables, and discard data of patients from the dataset who were missing these values.

Another major challenge in using the PRO-ACT database is that the time between clinic visits is not uniform across patients, or for a specific patient, and that the number of visits varies from patient to patient. In this research, we opted to use stationary modeling techniques (see Section 3.4) when experimenting to simplify modeling and data analysis. To do so, we extracted from the database only the first and last clinic visits recorded for each patient (along with the static data). In this manner, we were able to experiment with static models, while still addressing (in a simplified fashion) dynamic aspects of the data. The first and last clinic visits were selected because they each approximate a milestone in disease progression: the first clinic visit documents the patient at a time when they are healthiest (in the database) and closest to disease onset, while the last clinic visit documents the patient’s state at a time when they were most severely affected. Furthermore, these two clinic visits represent the maximal duration of follow-up reflected in the database, thus making prediction tasks the most difficult (and interesting).

Having completed the data-preparation phase, we created a uniform dataset of 3772 patients: our working dataset for experimentation and modeling.

### 3.3. Feature Selection

Another stage in data preparation is feature selection, aimed at reducing the problem dimensionality for the sake of model simplification and increasing efficiency and accuracy. For feature selection, we used the $J_{3}$ scatter criterion [33] that computes $trace(\Omega^{-1}B)$, where Ω is the within-class matrix, and *B* is the between-class matrix, which is a measure of class separability that is maximized for well-separated, compact feature-based class representations. We used the $J_{3}$ measure to compare sub-sets of a given size, and the accuracy of a classifier, which selects among the feature sub-sets of all sizes as selected by $J_{3}$ [11]. $J_{3}$ guarantees selecting the best (according to the scatter criterion for *k* features) sub-set of size k∈[1:K], and the classification accuracy guarantees selecting among all “best” sub-sets the sub-set with the “best" size. For each of the ten ALSFRS items, we implemented Algorithm 1 to determine the feature sub-set to be used when predicting the ALSFRS values:

**Algorithm 1:** Feature selection by the $J_{3}$ criterion [11]
**Input**: Potential features, *K* the maximal feature sub-set size desired, and ALSFRS target value
**Output**: Selected feature sub-set for the ALSFRS target value

**for** *k = 1:K* **do**



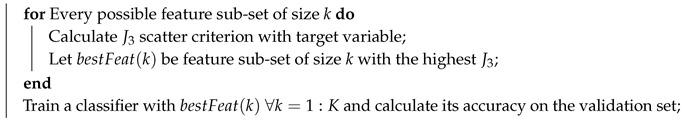



**end** (

Return: bestFeat(k) with the highest accuracy on the validation set


Table 2 summarizes the participation of laboratory variables in the selected lab test feature sub-sets for the different ALSFRS items when the classifier was a neural network (a two-layer perceptron with k/2 hidden units, when k<10, and 10 hidden units otherwise, trained using the conjugate gradient and backpropogation algorithms). All lab test results that were not discarded due to missing data (see Section 3.2) were included in the initial feature set, with K=25. Table 2 shows that the algorithm for feature selection favors feature sub-sets that include most of the laboratory variables. “Handwriting” is the exception to this, where the feature-selection algorithm chose a feature set with only four of the laboratory variables. Furthermore, we can see that three of the variables (bicarbonate, red blood cells, and white blood cells) were not selected in any of the feature sub-sets.

**Figure 3:**
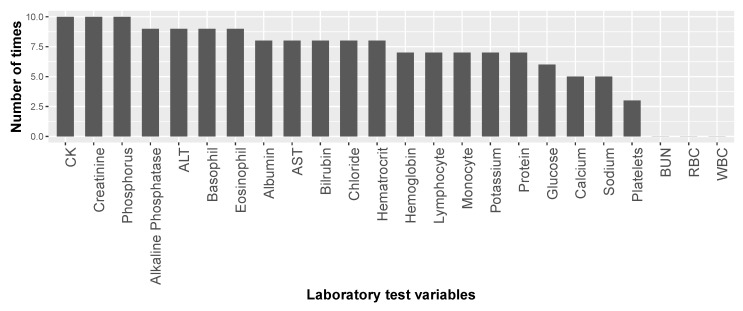
Number of times each of the laboratory test variables was selected in the feature sub-sets for the ten ALSFRS items.

Figure 3 presents the number of times each lab test variable was selected by the feature-selection algorithm among the ten ALSFRS items. We see that CK, creatinine, and phosphorus were selected in all ten cases, and four other laboratory test results were selected for nine ALSFRS items.

Table 2 and Figure 3 present the results of our first mechanism of feature selection that enables us to focus on the most important laboratory test variables with respect to ALS and to reduce the problem dimensionality before modeling. Then, while predicting ALSFRS disease state and identifying variable (factor) importance and their relations, our second mechanism of feature selection continues this process of dimensionality reduction (see below in Section 3.4.2).

### 3.4. Modeling

In this stage of the methodology, we first designed and trained models for ALSFRS prediction using either multi-class or ordinal classification algorithms (Section 3.4.1), and second performed knowledge representation using Bayesian networks (Section 3.4.2).

#### 3.4.1. Prediction by Multi-Class and Ordinal Classification

We design prediction models that classify disease state (the target variable is any of the ten ALSFRS items) at the last clinic visit recorded in the database. Before describing one of our main contributions, that of ordinal classification to ALS data, we wish to present the conventional multi-class classification, and for that, we use one of the simplest models, yet one of the most informative and interpretable ones, the decision tree (DT) [34].

We used DTs for predictive modeling for several reasons: (1) they are robust to outliers and noisy data, which are prevalent in our data; (2) they have the ability to find unrestricted hypothesis spaces, which allows us to find non-linear mappings from the feature space to the disease state; and (3) they are easy to interpret and extract further knowledge from, which is crucial in order that their results will be accepted by the medical community. We trained multiple classification trees, one for each of the ALSFRS items. We viewed the task as a five-class classification one (for ALSFRS values 0–4). The trees were trained using the C5.0 algorithm, where pruning parameters were determined empirically for each of the ALSFRS items separately using a validation set. Also, the more powerful random forest (RF) [35] and XGBoost [36] classifiers are actually based on multiple building of DTs, and the former is used as baseline in the ordinal classification experiments (Section 4.3).

Now, to focus on the task of ordinal classification with five categories, we experiment with three models geared towards ordinal classification: cumulative link models (CLMs) [37], which are extensions of generalized linear models (GLMs) for ordinal target variables, ordinal decision trees (ODTs), which are a class of models that represent a generalization of classification and regression trees (CART) [38] to ordinal target variables, and cumulative probability trees (CPTs) [39], which represent a simple method for extending classification to ordinal classification. The three algorithms are compared with a state-of-the-art conventional (non-ordinal) classifier, the RF classifier [35].

#### 3.4.2. Knowledge Representation by Bayesian Networks

Bayesian networks (BNs) use a graph-based representation as the basis for compactly encoding a complex distribution over high-dimensional spaces. In this graphical representation, nodes correspond to the variables in the problem domain, and the edges correspond to direct probabilistic interactions between the variables [40]. Over the past three decades, BNs have become a popular representation for encoding uncertain knowledge in expert systems [40,41]. They have several inherent advantages over other models [40,41,42]: (1) BNs allow for the manifestation and visualization of higher level interactions among variables; (2) when combined with Bayesian statistical techniques, they allow incorporation of prior domain knowledge, which is a valuable asset especially in medical domains; and (3) they compactly and intuitively encode knowledge, and are thus an excellent tool for knowledge extraction and representation where the trained model should be interpreted by medical clinicians.

In this study, we used BNs to explore the mechanisms underlying ALS and its progression. We used structure-learning techniques to learn the graph architecture directly from the data. This allowed us to further expose interesting relationships of the vital signs and lab test variables, among themselves and with the disease state (patient functionality).

For structure learning, we used an algorithm called risk minimization by cross validation (RMCV) [43]. While most algorithms for BN structure learning attempt to learn the graph structure from the data using some scoring function that is typically based on the likelihood of all graph variables given the data [42], the RMCV algorithm searches for the graph that maximizes the prediction accuracy using the class variable. This approach fits our domain very well, as we are attempting to learn a graph emphasizing variable relationships that have to do with the prediction of disease state, and not generally with the entire variable set. By using this approach, and maximizing the prediction accuracy of the learned structure with respect to disease state, we are focusing on variables influential to ALS, rather than attempting to maximize the likelihood for the entire (domain) variable set. Furthermore, by using RMCV, we are increasing the potential clinical benefit from using BNs, which are usually used only in knowledge representation, by enabling their use for predictive purposes as well as for knowledge representation and explanation.

After discretizing continuous variables in our database (RMCVs, as most BNs, work on discrete variables) using the minimum description length supervised discretization algorithm [44], we learn from the data a BN using the RMCV algorithm. We initialized the RMCV search with an empty graph. As was outlined in the Introduction section, we repeated this procedure for each ALSFRS item to offer knowledge representation and to demonstrate interrelations among variables for each patient functionality separately, expanding our capability to understand different mechanisms of the disease.

### 3.5. Experimentation

In accordance with the Introduction section, the goals of the experiments were: (1) to construct models that can accurately map important vital signs and laboratory test variables to disease state measured at present or in the future, additionally gauging the effect of adding time-dependent information on model performance; (2) to propose and test algorithms that let the ordinal information contained in the disease state to augment learning to account differently for different prediction errors; (3) to find important variables that can accurately predict and informatively explain the disease state, and to construct models that can depict how these variables influence disease state and are related to each other; and (4) to apply previous goals to each patient functionality separately to help focus the clinical diagnosis of disease progression on different mechanisms of the disease.

To this end, we trained ML models in three different ”semi-temporal” settings for each of the ALSFRS items using both multi-class and ordinal classification algorithms. In each, the models are trained to predict the ALSFRS values at the time of the last clinic visit for the patients.

**Setting 1** (“Last”): The models are based only on the physiological and lab test (static and temporal) variables collected during the last clinic visit. In this setting, the models can be thought of as attempting to classify disease state at present based only on current information. Our main goal in this setting is to gauge the potential of the data contained in the PRO-ACT database to explain and predict the disease state.

**Setting 2** (“Both”): The models are fed also variables collected at the first clinic visit, as well as an additional variable, which represents the amount of time (in days) that has passed between the first and last clinical trials. We can think of the models in this setting as attempting to classify disease state in the present, but this time using information from the past as well, gauging the potential value of adding time-dependent information to the learning procedure.

**Setting 3** (“First”): The models are trained using only the data collected during the first clinic visit. Here, the same variable that represents the number of days passing between the first and last clinic visits, representing the number of days in the future for which we would like the models to predict the disease state, is inputted the model. In this setting, the models are attempting to predict disease state at a certain point in the future. This task is the most difficult of the three, as the learners need to use very early information about patients to predict their disease state far in the future (Please note that the mean and standard deviation values of the interval between the first and last visits are 333.8 and 167.5 days, respectively).

In each of the settings, the models attempted to classify disease state for each of the ALSFRS items separately and to check differences among different patient functionalities, so in effect each setting comprises learning ten different models.

It is important to mention that we did not include the ALSFRS values from the first clinic visit as variables for learning in either of the settings, even though they were “available” in the last clinic visit. This was done intentionally, so as to maximize the requirement of the mapping from physiological and laboratory variables to disease state, rather than just letting the existing ALSFRS values predict future ALSFRS values and dominate the models’ learning procedure, “overshadowing” the physiological and laboratory data, their interrelations, and their relations to, and impact on, the disease state. This is in contrast to past research that attempted prediction of disease state or progression rate by incorporating the current values of ALSFRS into the training data (e.g., [27]).

Besides considering the target variable as discrete with five classes, we also enabled classification models to exploit ordinal information, e.g., that predicting a patient’s walking functionality is 3, while it is actually 1, is harsher than if predicting it as 2, and thereby to penalize miss-predictions differently (in this example, the former prediction should be penalized more than the latter one).

We executed a CV-10 procedure using a single fold as a test set and the remaining nine as a training set, and compute and report mean classification accuracy (%) (for the DT) and mean absolute error (MAE) (for the ordinal classifiers) on the ten test sets, so that model performance can be inspected and evaluated on previously unseen data representing future patients. Though ordinal classification and MAE are the natural setting and performance metric, respectively, reporting classification accuracy provides a baseline for the case where accounting for error severity is less important.

## 4. Results and Analysis

Following the partition of the experiments to those for testing prediction and those for testing knowledge representation, we also partitioned the Results section accordingly.

### 4.1. Multi-Class Classification

While the output of the multi-class prediction models in classifying a disease state is the classification accuracy, that of ordinal prediction models is the (mean absolute) error. Starting with our multi-class model, the DT, Table 3 shows that its classification accuracy on the test set is high in all three settings. Since the classification task has five classes, random selection would likely yield approximately 20% classification accuracy, yet the DTs yield between 71% and 84.7%, depending on the task setting and ALSFRS item.

Also, we can see trends in the classification accuracy. For each of the ALSFRS items, we can see that the classification accuracy is improved when adding information from the first clinic visit (i.e., moving from Setting 1 to 2) and reduced when attempting to predict future disease state (i.e., moving from Setting 1 to 3).

However, classification accuracy alone is not enough to establish that the models are performing well in their designated tasks. We would like to establish that the models are not simply classifying to the most frequent categories, and thus achieving the relatively high classification accuracy. To this end, we inspected the confusion matrices deriving from the models’ predictions.

**Figure 4:**
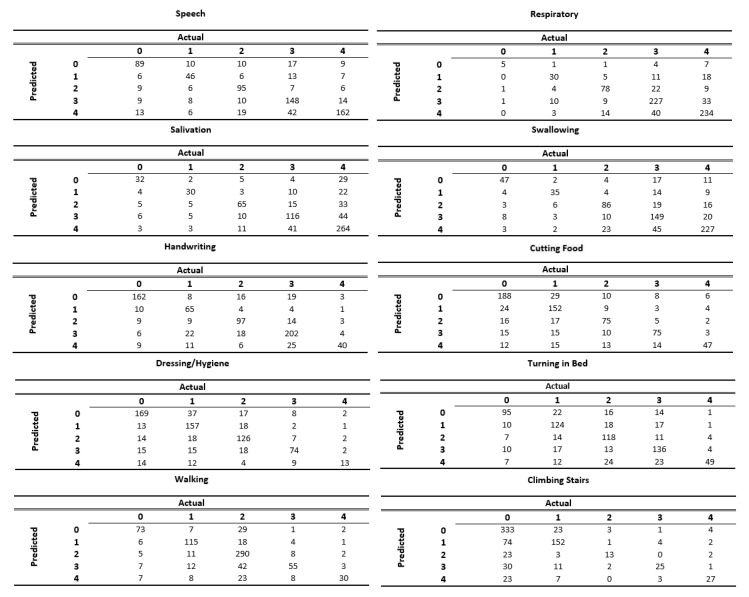
Confusion matrices for each of the ALSFRS items when predicting disease state using only information from the first clinic visit.

Figure 4 displays the confusion matrices for each of the ALSFRS items. These matrices represent the output of the models from the most difficult task (i.e., Setting 3). We can see that the models succeed in classifying patients to less common categories as well as to the most frequent category. For instance, in the ALSFRS function “Respiratory”, which is heavily skewed towards Categories 3 and 4, we can see that the model correctly classifies five out of the seven patients in the test set who had a value of 0. We can also see that some models (e.g., those for “Salivation” and “Swallowing”) excel in predicting “severe” patients (having ALSFRS values of 0 and 1), whereas other models (e.g., those for “Walking” and “Climbing Stairs”) are more accurate in predicting “mild" patients (having ALSFRS values of 3 and 4). Furthermore, we can see that values in the confusion matrices tend to group along the main diagonal (which represents correct classifications) far more than off of it.

### 4.2. Ordinal Classification

Assume *X* and *Y* are the predicted and observed vectors for *n* records, respectively, the MAE is the average of the absolute error between prediction and true values, i.e., $MAE=\sum_{i=1}^{n}\left|y_{i}-x_{i}\right|/n$. Table 4 details MAE results for the three settings and three ordinal prediction models in comparison to a RF to see if accounting for the ordinal nature of the target variable can improve performance. Table 4 shows that the average MAE is between ≈0.6 and ≈2 in points of ALSFRS scores depending on the model, setting, and function. Statistical testing reveals that there is no significant difference between the performance of CLM and ODT (*p*-value ≈0.39 for a paired student’s *t*-test) in the first and second settings and both err in less than a point in most cases. In the predictive setting (setting 3), the difference between them is significant (*p*-value ≈0.012) in favor of ODT. However, in all three settings, there are significant differences (*p*-value ≈0) between CLM and CPT, CLM and RF, ODT and CPT, and ODT and RF. The difference between RF and CPT is significant (*p*-value ≈0.026) in favor of CPT for all three settings, but depending on how we account for multiple testing, this might not be considered significant.

Table 4 shows that accounting for the ordinal nature of the problem clearly improves prediction performance, and CLM and ODT significantly outperform the RF classifier, which can typically be expected to achieve performance at least as good as a tree model. The table also shows that, as expected, the predictive setting poses more difficulty than Settings 1 and 2; all of the models (with the exception of CPT) perform significantly better in Settings 1 and 2 than in 3. Further investigation reveals that the CPTs are simply predicting the class with the highest a-priori probability, and therefore performance is not improved between the settings for this model. Finally, using CPT models as a baseline, we can see that all the other models are able to improve prediction performance as measured by MAE significantly over a model that simply predicts the class with the highest a priori probability.

### 4.3. Knowledge Representation and Explanation

A significant goal of this research was the exposure and explanation of important mechanisms underlying the disease. In this respect, one aim is to identify variables important to ALS prediction (Section 4.3.1), and here, it can be argued that prediction (i.e., Setting 3; ”First”) is the most interesting (and most difficult) task. However, for the explanatory aim, in order to identify variable interrelations and their relations to the disease state (Section 4.3.2), we analyzed the models constructed using both clinic visits (Setting 2; ”Both”), as these together are the most suitable for identifying relations between variables that are measured close to onset and those reflecting later stages of the disease. Later, based on these identifications, we can detect and analyze value combinations of important variables that characterize patient populations of interest, e.g., severe or mild (Section 4.3.3).

#### 4.3.1. Decision-Tree Based Explanation

For each ALSFRS item, predictor importance is calculated by computing the reduction in variance of the target (class) variable due to the predictor via a sensitivity analysis [45]. After ranking this reduction (importance), we can examine the average predictor importance over all ALSFRS models, or that for each function separately.

First, a sensitivity measure of variance is computed for each predictor

$$S_{i}=\frac{V_{i}}{V(Y)}=\frac{V(E\left[Y|X_{i}\right])}{V(Y)},$$

where V(Y) is the unconditional target variance,

$$V_{i}=V\left(E\left[Y|X_{i}\right]\right)=V\left(Y\right)-E\left[V\left(Y|X_{i}\right)\right]$$

is the conditional target variance on variable $X_{i}$, and the multi-dimensional distributions are approximated e.g., via Markov chain Monte Carlo (MCMC) methods. Then this predictor importance is normalized for each of the *k* variables, e.g.,

$$VL_{i}=\frac{S_{i}}{\sum_{j=1}^{k}S_{j}}.$$

Finally, the normalized sensitivities of the predictors ($VL_{i}$) are ranked, determining an order of importance for the predictors [45] by which they can be optimally selected for classification.

**Figure 5:**
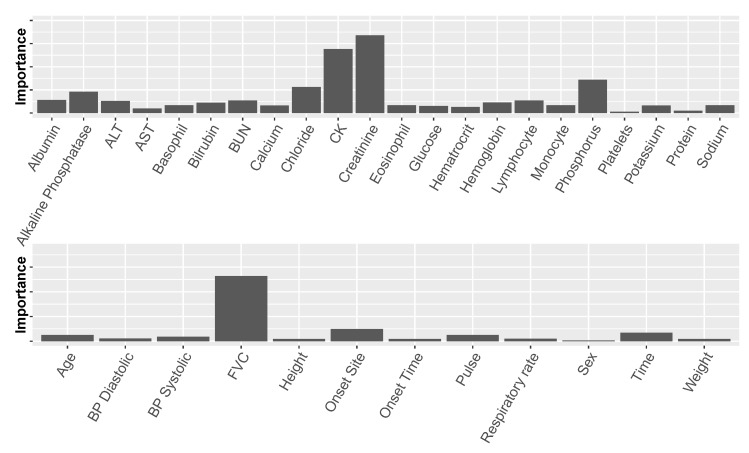
Average predictor importance for laboratory test variables (**top**) and non-laboratory test variables (**bottom**). “Time” measures the time values were documented from the beginning of a clinical trial. BP represents blood pressure.

Similar to our feature-selection mechanism (Section 3.3) that chose the best set of variables with respect to set size and content, this measure of predictor importance is highly valuable, as it allows us, together with the former mechanism, to analyze the models and find predictors that are potentially significant with regard to the disease. Figure 5 displays the predictor importance based on the DTs for the laboratory (top) and non-laboratory (bottom) variables averaged over the ten ALSFRS items and two instantiations of each variable from data of the two clinic visits (Setting 2).

The non-laboratory variables that proved important: FVC, Onset Site, and Time, are also known to be important from previous research [1,4,27,28]. The most important of these is FVC, which is a symptom of the disease as well as a predictor. While non-laboratory variables may be important with regard to prediction, they are of less interest when exploring underlying mechanisms of the disease and risk factors. Therefore, in this study, we were more interested in analyzing laboratory variables.

Figure 5 (top) shows that among the laboratory variables, there are several predictors that stand out, namely creatinine (a naturally occurring nitrogenous organic acid involved in adenosine triphosphate (ATP) production), CK (creatine kinase, an enzyme found e.g., in skeletal muscles), chloride, phosphorus, and alkaline phosphatase. Creatinine, CK, and phosphorus have recently been mentioned in other studies as being related to the disease (creatinine and CK were also found to correlate with each other) [27]. To the best of our knowledge, this is the first time that the rest of these predictors are identified as being related to ALS.

**Figure 6:**
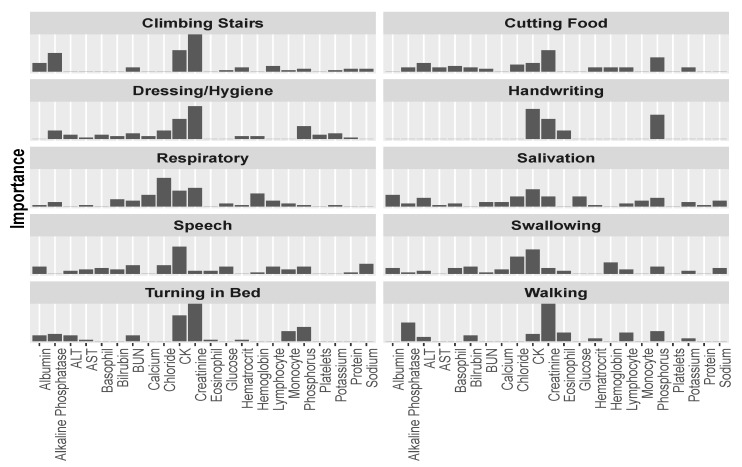
Predictor importance among the laboratory variables for each of the ALSFRS items.

Further exploring predictor importance, we can “drill down” in our analysis and inspect this importance with relation to a specific ALSFRS item, rather than averaged over the ten values. When visualized thus (as in Figure 6), it is clear to see how different aspects of the disease relate differently to the laboratory variables. This leads us to believe that there might be justification for viewing the different aspects of the disease separately, rather than as a sum (or average) over all of the functions, as is the tendency of past research.

In Figure 6, we can see that CK is the most or second most important factor for most ALSFRS items, and especially for bulbar functions (Swallowing, Speech, and Salivation), but also for full body functions (Dressing/Hygiene and Turning in Bed) and other functions (e.g., Handwriting and Climbing stairs). Creatinine is the most important predictor for functions that are related to major muscles of the lower body, such as Walking and Climbing Stairs, or in full body functions, such as Dressing/Hygiene and Turning in Bed. Chloride is the most important predictor for the Respiratory function (a function that plays an extremely important role in disease progression [1]), and the second most important predictor for Swallowing. Figure 6 also demonstrates that the contribution to the average importance of phosphorus (Figure 5) mainly involves upper and full body functions: Cutting Food, Handwriting, Dressing/Hygiene, and Turning in Bed, and alkaline phosphatase mainly relates to Walking and Climbing Stairs.

Realizing that certain variables jointly show important relationships to certain ALSFRS items, while not to others, led to the idea of clustering the ALSFRS items themselves into higher level groupings.

**Figure 7:**
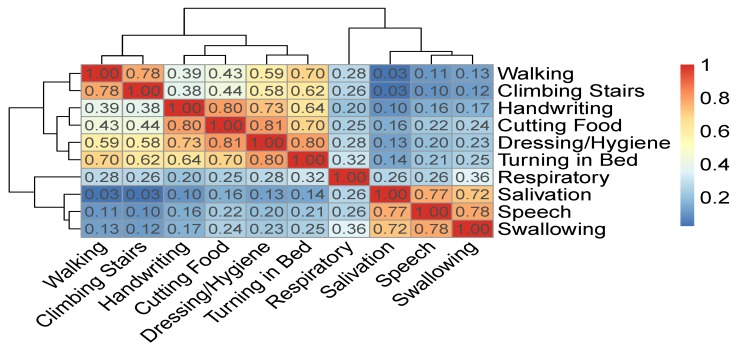
Correlation matrix and hierarchical clustering of ALSFRS items.

Figure 7 displays a correlation matrix between the ALSFRS items and a dendrogram that results from applying hierarchical clustering by average linkage to the pairwise correlation values between the ALSFRS items. Both the correlation values (represented by a colormap) and the dendrogram show that the ALSFRS items can be divided into five groups: (1) Salivation, Speech, and Swallowing, (2) Handwriting and Cutting Food, (3) Walking and Climbing Stairs, (4) Turning in Bed and Dressing/Hygiene, and (5) Respiratory. Thus, it is clear that higher level groupings of the ALSFRS items can be justified. Furthermore, these higher level groupings are semantically interpreted (see Table 5) and aligned with medical convention [1,2,4], seeing the five groups as related to bulbar, upper limbs, lower limbs, full body, and respiratory function, respectively. It is interesting to see that if we break up Group 4 that describes full body functioning, such that Turning in Bed is linked to the lower limbs group and Dressing/Hygiene is linked to the upper limbs group, we get another common medical ALSFRS grouping [20].

**Figure 8:**
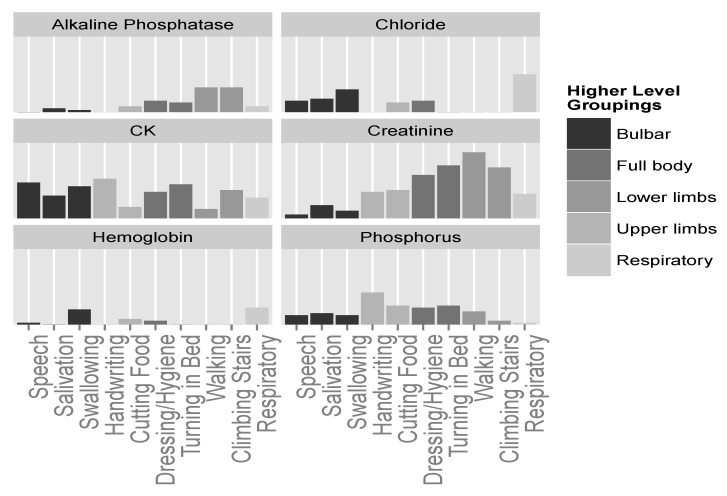
Predictor importance of significant laboratory test variables for the different ALSFRS items. Higher level groupings color the ALSFRS items.

Continuing this line of exploration, we can now plot the importance of strong predictors mentioned above, and see whether their importance to the different ALSFRS items is in agreement with the groupings mentioned. Figure 8 shows that some of the laboratory variables, such as creatinine and CK, seem to be important for many functions. Even between these two, we can see that, similar to Figure 6, creatinine seems to have a stronger relationship with lower limbs and full body actions, and much less of an association with bulbar functions. CK is a more meaningful predictor of the bulbar and full body functions. CK levels for some upper limbs functions (e.g., Handwriting) are similar to those of some lower limb functions (e.g., Climbing Stairs), and indeed other studies [46] found no significant difference between these two levels. Yet, the mechanism of CK elevation in ALS is not clear. It may be due to changes in the metabolism in the damaged muscles, increase in the muscle cell membrane permeability [47], or up-regulation of this enzyme to provide an energy substrate in a hypercatabolic condition [46].

Phosphorus seems to be more connected with upper limbs and full body functions (see also Figure 6), and chloride shows importance only in relation to bulbar and respiratory aspects of the disease. Finally, alkaline phosphatase and hemoglobin, while seemingly less important predictors, show relationships with specific groupings; alkaline phosphatase seems to be connected with lower limbs; and hemoglobin seems to be connected with swallowing and respiratory functions, although to a weaker extent.

#### 4.3.2. Bayesian Network-Based Explanation

Analyses of the supervised classification models have helped to pinpoint important physiological and lab test variables, and to map them to different aspects of the disease. However, this analysis is limited in that we cannot see interactions between these predictors or understand context and flow of influence within the models. Fortunately, BNs allow us to model the problem in such a way that exposes higher level interactions and relationships between variables, and between them and the target variable.

To this end, we learn BNs from the data using the RMCV algorithm (Section 3.4.2) that is initialized from the empty graph. We learn one network for each of the ALSFRS items. Besides improved accuracy compared with other BN learning algorithms, using the RMCV algorithm while defining the class variable to be the ALSFRS value restricts the learning process to concentrate on relationships and interactions that are important with respect to the disease state itself (Section 3.4.2). To avoid the curse of dimensionality and to facilitate learning, we initialized the RMCV algorithm using only variables measured in the last clinic visit.

**Figure 9:**
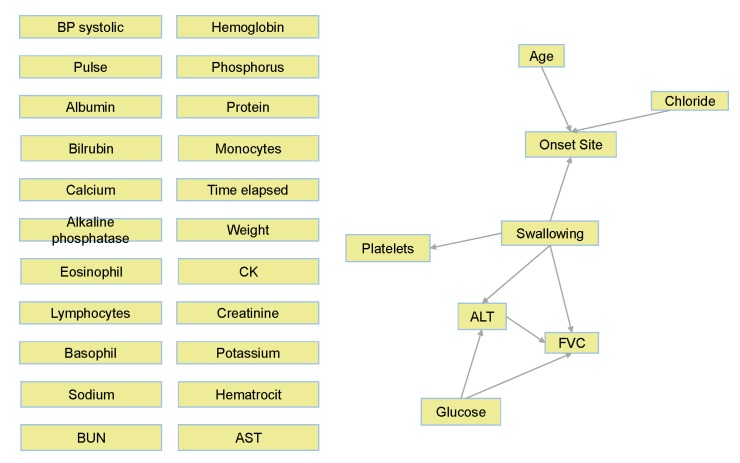
Complete BN graph for Swallowing.

Figure 9 displays the complete BN learned for the ALSFRS item “Swallowing”. We can see that of the entire variable set, only relationships and connections that were important with respect to the class variable (Swallowing) were learned, where unimportant (according to the RMCV) variables are not connected to the graph. This is another advantage of the RMCV algorithm, in that learning the BN graph (structure) also applies naturally to feature selection that is augmented towards prediction. After learning is complete, only the Markov blanket (MB) [40]—the variable, its parents, children, and children’s co-parents— for the class variable is kept, as only variables in the MB can affect and be affected by the class variable (see the right side of Figure 9).

**Figure 10:**
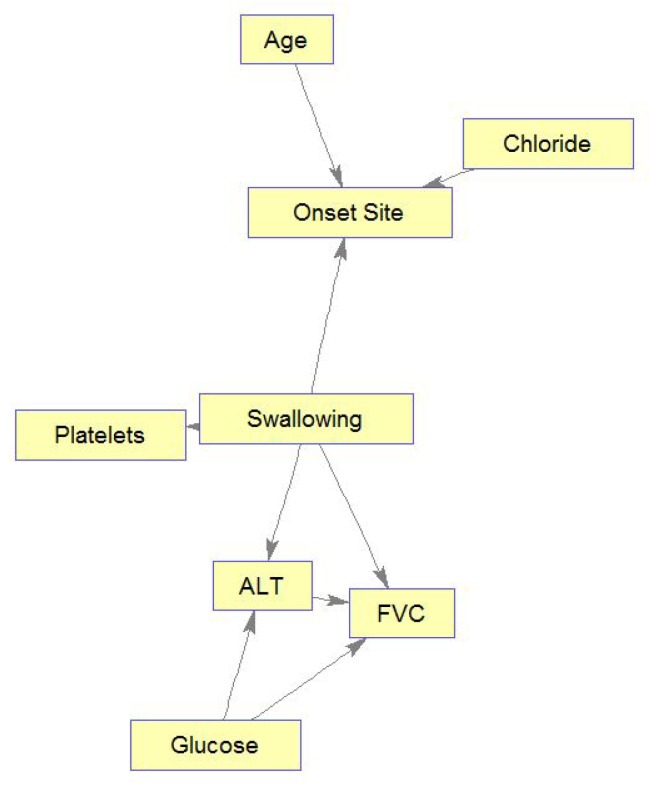
Markov blanket for Swallowing.

Figure 10 shows only the MB for Swallowing (similar MBs were learned to all ten ALSFRS items). This figure demonstrates another advantage of BNs, which is their interpretability. Based on these graphs, ALS clinicians are able to explain connections they see in the BN from their own knowledge, even though these sometimes may not have been known or thought of in advance. Alternatively, medical knowledge can validate the BN model. For example, the connection seen in Figure 10 between Swallowing and Onset Site (Swallowing→Onset Site) is well known, as almost all bulbar-onset patients develop excessive drooling due to difficulty in swallowing saliva [48]. FVC, which is a measure of the respiratory system, is also affected by the ability to swallow, and difficulties in swallowing worsen the ability of the respiratory system (see the directed edge Swallowing→FVC in Figure 10). Low glucose levels in the blood may be caused by difficulties in swallowing (low food intake), and these low levels may cause harm to the breathing muscles, which decreases FVC (see directed edge glucose→FVC). While the connection Glucose→ALT (alanine aminotransferase) may represent the Glucose-Alanine cycle between the muscles and the liver, the connection Swallowing→ALT←Glucose represents that when the value of ALT is known, Swallowing and Glucose are (conditionally on ALT) dependent. Note also that elevated levels of ALT in the blood that are measured with elevated levels of CK (although CK is not part of Swallowing’s MB, it is an important predictor of Swallowing; see Figure 8) is a major indicator of ALS [48], as both enzymes are included among the muscle enzymes [49]. In addition, studies show that Age does not affect ALS, despite the higher frequency of bulbar-onset cases among older women (see directed edge Age→Onset Site), but the complex relationships between age and bulbar onset remain to be clarified [50]. Furthermore, it is possible that the diet of the bulbar-onset patients, having difficulties with swallowing, and thus moving to tube feeding, is not balanced with respect to chloride (see the edge Chloride→Onset site). However, this idea requires further investigation.

**Figure 11:**
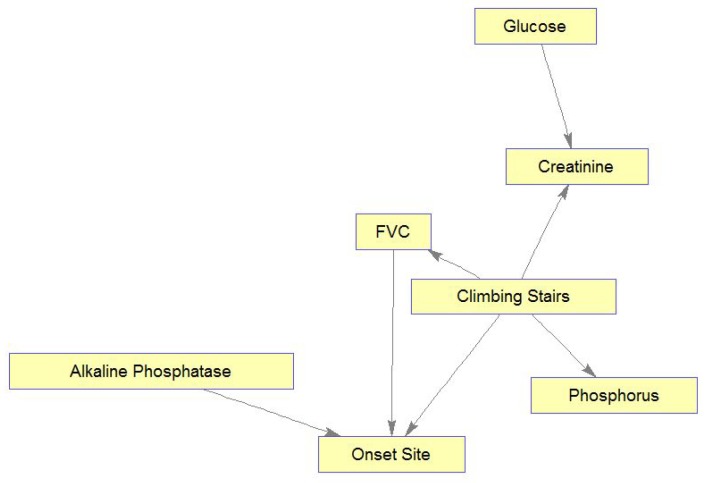
Markov blanket for Climbing Stairs.

Similarly, Figure 11 shows the learned MB for Climbing Stairs, demonstrating interesting relations among the MB’s variables. Figure 11 shows relations of patient functioning ability in climbing stairs with four lab test results, FVC, and onset site. Also, these relations can mostly be explained medically. Briefly, as glucose and creatinine are important for and related to energy metabolism, they are connected by an edge. Glucose is the main energy source of muscles and is needed to create and maintain muscle activity, while serum creatinine diminishes with disease progression following a decrease in the muscle mass [51]. Please note that if the muscle mass was measured (or estimated) in the clinic visits and introduced into the model, more interesting connections among Glucose, Creatinine, Climbing Stairs, and muscle mass could have been identified. Phosphorus changes are related to muscle weakness that is experienced while climbing stairs. High alkaline phosphatase values are found in people with low motor performance, due to physical disability (e.g., in climbing stairs), and in our case, these people may be limb-onset patients (Onset Site = limb). Respiratory insufficiency, which is reflected in low values of FVC, makes any physical activity, e.g., climbing stairs, much more difficult. Note again that if information about muscle condition could be brought into the model through some “muscle variables”, these variables could be found related to both the abilities to climb stairs and to breathe (affecting FVC).

#### 4.3.3. Analysis of Variable Value Combinations

Based on the MBs, we analyzed distributions over value combinations of important variables included in the MB with respect to the different aspects of the disease (ALSFRS items). If the MBs were small enough, we could simply analyze combinations for all variables in the MB of each function. However, since nearly all BNs yielded moderate-sized MBs, which are intractable to analyze in this manner, we incorporated knowledge from our own predictor importance analysis (Figure 6) to derive sets of four important variables selected from the MB for each function. Table 6 shows these four-variable sets for the ten ALSFRS items.

**Figure 12:**
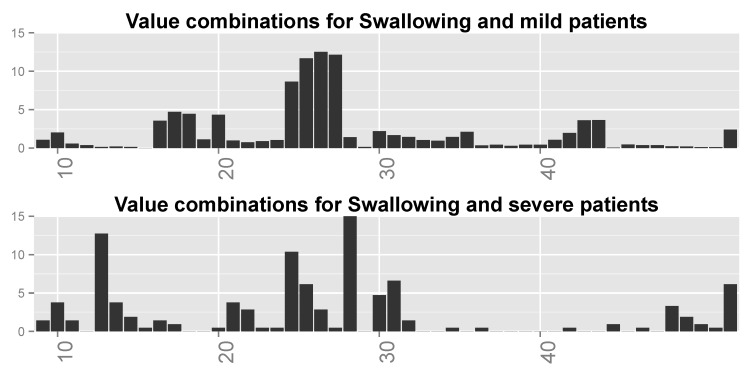
Distributions over all possible (64) value combinations of the four important variables for Swallowing for (**top**) mild patients and (**bottom**) severe patients. Numbers along the *x*-axis are indices of the combinations.

We inspected the different combinations of the important variables for each function (ALSFRS item) and divided the patient population into two groups: those with an ALSFRS value of zero or one during the last clinic visit (“severe” patients), and those with an ALSFRS value of 3 or 4 (“mild” patients). We then computed for each ALSFRS item the frequencies of all possible combinations of the four variables for each group. These frequencies are plotted in Figure 12 for Swallowing (and computed also for all other ALSFRS items), demonstrating significant differences between severe and mild patients. Some of the value combinations are very frequent for mild patients while they are less frequent or even rare for severe patients and vice versa.

**Figure 13:**
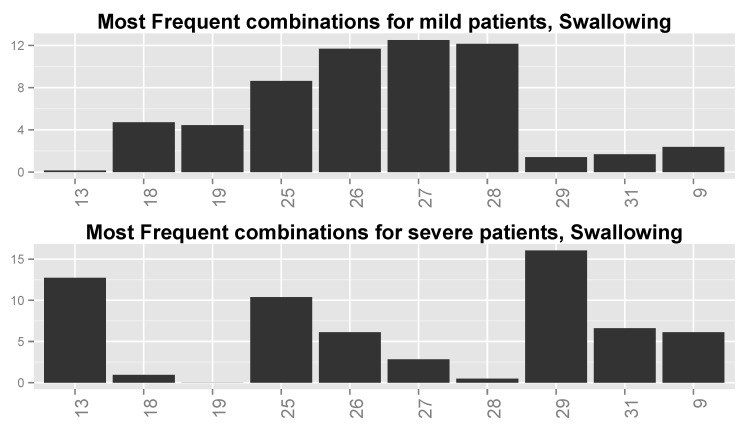
Distributions of most frequent combinations for Swallowing for (**top**) mild patients and (**bottom**) severe patients. Numbers along the *x*-axis are indices of the combinations.

Finally, we inspected the six most frequent value combinations for both groups of patients for every function. Table 7 shows these combinations for the four most important variables for Swallowing (Table 6), together with the frequencies of severe and mild patients for each combination. Combinations 1–6 are the most frequent for severe patients, while combinations 3 and 6–10 are the most frequent for mild patients (note that combinations 3 and 6 are shared by the two patient groups). Figure 13 shows the frequencies of these value combinations, demonstrating visually the difference between mild and severe patients with respect to the value combination frequencies. Please note that tables such as Table 7 and graphs similar to that in Figure 13 for the rest of the functions show that changes in the values of at least a single variable are responsible for the differences in frequencies between severe and mild patients.

We can see from Table 7 and Figure 13 that the combinations are different between the two groups of patients. For example, the combinations for which FVC = low (including combination 4 for which FVC = moderate–high) and ALT = high, regardless of the values of Onset Site and Chloride, are typical to severe patients, sometimes up to an order or two in magnitude more than for mild patients. Combinations 6–10 and 3 with Onset Site = limb, Chloride = normal, and FVC = not low (except for combination 3), regardless of the value of ALT, are prevalent in mild patients even up to two orders in magnitude more than for severe patients. The two combinations that are frequent for both patient groups (i.e., 3 and 6) have frequencies that are not very different between these groups. It might be that the second group (limb-onset patients with FVC values that are not low) is of patients in the database that are in their early stages of disease and thus, with high probability, these are mild patients, whereas the first group (patients with low FVC values) is of patients in their late stages of disease, and thus, with high probability, severe patients.

We can see that this type of analysis of the distributions of value combinations of important predictors has the potential to expose and explain interesting and possibly meaningful underlying mechanisms of ALS.

## 5. Conclusions

One contribution of this study with regard to prediction models is the construction of well-performing models that make no assumption regarding disease progression behavior and are able to effectively map from physiological and laboratory information directly to disease state at some given time in the future. Another contribution of this study is the exploitation of ordinal classification models to improve predictive performance, something that, to the best of our knowledge, has not been demonstrated in past research.

Furthermore, past research has mainly viewed the disease state as a sum of the ALSFRS items. In this study, we demonstrated the value of modeling the disease aspects separately (as depicted by the individual items). By doing this, we could map specific variables as being important with regard to certain aspects of the disease, while demonstrating little or no relation to others.

Another contribution of this study is an in-depth analysis of potentially important physiological and laboratory variables. We used ML techniques to identify such variables, some of which have not been previously identified in the literature as being related to ALS. Among these are creatinine, CK, and phosphorus, which were only recently identified as related to the disease [27,46,47], and chloride, alkaline phosphatase, and others, which are, to the best of our knowledge, not currently identified with ALS.

Following this logic, we showed that the ten ALSFRS items can be statistically grouped into four or five easily interpretable higher level groupings. We then showed that certain predictors map well to specific groupings, while less so to others. For instance, alkaline phosphatase, which does not demonstrate a very strong relation to the sum of the ALSFRS items (Figure 5), appears to be relevant to the deterioration of lower limbs functionality (Figure 8) probably due to its relation to muscle metabolism.

Another contribution of this study is the use of graphical models, namely Bayesian networks, for explanatory purposes. We showed that modeling the disease as a set of probabilistic graphical models holds potential for both explanation and interpretation of disease mechanisms, as well as for the identification of important variables via the learned Markov blanket. We concluded the analysis by using the information from the MBs to run an analysis of variable value combinations that sheds more light on the interactions and relationships between the important variables and patient functioning for different groups of patients.

One clinical aspect of our study is the ability of our algorithms to provide the physician, patient, and caregiver detailed predictions of disease state and rate for each type of functionality, and also tools for risk factor and biomarker investigation for the pre- and post-symptomatic disease stages, respectively. We believe that these algorithms, when implemented and adopted clinically, could reduce uncertainty and improve the quality of life of patients and caregivers. If we can predict, for example, that a patient’s walking or speech ability will deteriorate in six months, he or she can organize the home to address their needs or move to a more appropriate environment, or start looking for a specific device to communicate with people. This will also enable physicians to know where to begin specific treatment, and whether and when to focus on the respiratory system or physiotherapy.

In a step toward accomplishing that, our algorithms, although trained using the PRO-ACT database, have recently been successfully validated using data collected over more than 20 years in a large ALS clinic. This success further pushes us to believe that the application of the algorithms also in other clinics or using other databases, although this will require some tuning and retraining, may be found successful too. In addition, we recently developed and implemented our algorithm in an information system, and installed it in a large ALS clinic, with the hope that it will advance daily clinical practice in ALS. Currently, we are also moving the system to the cloud, which will further facilitate its use globally.

It is our desired hope that the approaches brought in this study will lead to new medical research opportunities and insights into the treatment of patients with ALS.

## Figures and Tables

**Table 1:** Summary of data extracted from the PRO-ACT database and used in this study.

Data Table	Description
ALSFRS	ALS functional rating scale values
Demographics	Demographic data, such as gender, age ${}^{a}$, ethnicity, etc.
FAMHX	Family history concerning neurological diseases
FVC	Forced vital capacity (FVC) test values
Riluzole	If patient is taking Riluzole (the leading drug for ALS) or not
Patient ALSHX	Patient’s disease history: time of onset ${}^{b}$, symptoms at onset, etc.
Vitals	Vital signs throughout the trials
Labs	Multiple laboratory test results throughout the trials

${}^{a}$ Age denotes the age of a patient at the time the clinical trial began; ${}^{b}$ Time of onset, referred to as onset time in this study, is documented relative to the time of clinical trial initiation.

**Table 2:** Summary of lab test feature selection for each ALSFRS item.

		Speech	Salivation	Swallowing	Handwriting	Cutting Food & Eating	Dressing/Hygiene	Turning in Bed	Walking	Climbing Stairs	Respiratory
	Basophil	✓	✓	✓		✓	✓	✓	✓	✓	✓
	Eosinophil	✓	✓	✓	✓	✓	✓	✓	✓	✓	✓
	Lymphocyte	✓	✓	✓		✓			✓	✓	✓
	Monocyte	✓	✓	✓			✓	✓		✓	✓
	Albumin	✓	✓	✓		✓	✓	✓		✓	✓
	Alkaline phosphatase	✓	✓	✓		✓	✓	✓	✓	✓	✓
	ALT	✓	✓	✓		✓	✓	✓	✓	✓	✓
	AST	✓	✓	✓		✓	✓	✓	✓	✓	✓
	Bicarbonate										
	Bilrubin	✓	✓	✓		✓	✓		✓	✓	✓
	BUN	✓	✓	✓		✓	✓	✓	✓	✓	✓
	Calcium	✓	✓	✓		✓	✓	✓		✓	✓
	Chloride	✓	✓	✓		✓	✓	✓	✓	✓	✓
Laboratory Variables	CK	✓	✓	✓	✓	✓	✓	✓	✓	✓	✓
	Creatinine	✓	✓	✓	✓	✓	✓	✓	✓	✓	✓
	Glucose	✓				✓	✓			✓	✓
	Hematrocrit		✓	✓		✓	✓	✓	✓	✓	✓
	Hemoglobin	✓	✓	✓		✓	✓			✓	✓
	Phosphorus	✓	✓	✓	✓	✓	✓	✓	✓	✓	✓
	Platelets					✓	✓				
	Potassium		✓	✓		✓	✓		✓	✓	✓
	Protein	✓	✓	✓		✓	✓	✓		✓	✓
	Red blood cells										
	Sodium	✓	✓	✓			✓	✓			✓
	White blood cells										

**Table 3:** Accuracy of DTs for the three settings (only last clinic visit, both, and only first clinic visit). Values depict percentages of correct classification for the different settings and ten ALSFRS items.

Setting	Speech	Salivation	Swallowing	Handwriting	Cutting Food and Eating	Dressing/Hygiene	Turning in Bed	Walking	Climbing Stairs	Respiratory
Last	81.4	82.0	82.2	75.0	78.8	80.1	77.3	79.2	82.3	82.3
Both	82.5	84.7	82.4	80.1	79.7	83.8	82.9	82.3	83.7	83.2
First	79.5	75.9	78.3	71.0	71.6	78.8	73.3	78.4	77.8	74.5

**Table 4:** MAE averaged over CV-10 for three settings, four prediction models, and ten ALS functions (**bold** is best over algorithms).

	Alg.	Speech	Respiratory	Salivation	Swallowing	Handwriting	Cutting Food	Dressing/ Hygiene	Turning in Bed	Walking	Climbing Stairs
Last visit	CLM	**0.78**	0.67	0.81	**0.76**	1.07	1.05	**0.82**	**0.85**	**0.80**	0.74
	ODT	0.79	**0.66**	**0.78**	0.77	**1.02**	**0.99**	0.84	0.89	**0.80**	**0.73**
	CPT	1.77	2.11	2.02	2.00	1.46	1.25	1.13	1.33	1.20	0.91
	RF	1.37	1.42	1.57	1.45	1.44	1.23	1.09	1.22	1.14	1.02
Both visits	CLM	**0.71**	**0.62**	**0.76**	**0.68**	1.06	1.02	**0.81**	**0.84**	**0.77**	**0.76**
	ODT	0.78	0.63	0.79	0.74	**1.01**	**1.00**	0.85	0.89	**0.77**	0.77
	CPT	1.76	2.13	2.02	2.00	1.47	1.29	1.12	1.39	1.24	0.94
	RF	1.39	1.39	1.61	1.46	1.45	1.20	1.10	1.23	1.14	1.01
First visit	CLM	1.55	0.89	1.15	1.20	1.42	1.50	1.37	1.39	**0.85**	**0.95**
	ODT	**1.16**	**0.77**	**1.01**	**0.99**	**1.22**	**1.15**	**1.02**	**1.10**	**0.85**	0.96
	CPT	1.77	2.11	2.01	1.96	1.45	1.52	1.41	1.30	1.17	0.96
	RF	2.18	1.61	2.06	2.03	1.84	1.26	1.20	1.60	1.34	0.99

**Table 5:** A higher level grouping of ALSFRS items based on the dendrogram of Figure 7 as is interpreted by medical convention.

Group	ALSFRS Functions	Semantic and Medical Interpretation
1	Salivation, Speech, Swallowing	Bulbar
2	Handwriting, Cutting Food and Eating	Upper limbs
3	Walking, Climbing Stairs	Lower limbs
4	Turning in Bed, Dressing/Hygiene	Full body
5	Respiratory	Respiratory

**Table 6:** Four important variables selected from the MB for each ALSFRS item.

Function	Variable 1	Variable 2	Variable 3	Variable 4
Speech	FVC	Onset Site	CK	chloride
Salivation	FVC	Onset Site	creatinine	potassium
Swallowing	FVC	Onset Site	ALT	chloride
Handwriting	FVC	Onset Site	CK	potassium
Cutting Food	FVC	CK	chloride	phosphorus
Dressing/Hygiene	FVC	Onset Site	potassium	CK
Turning in Bed	FVC	Onset Site	creatinine	potassium
Walking	FVC	alkaline phosphatase	creatinine	CK
Climbing Stairs	FVC	alkaline phosphatase	creatinine	phosphorus
Respiratory	FVC	CK	hemoglobin	Potassium

**Table 7:** Six most frequent value combinations for severe and mild patients for Swallowing (note that combinations 3 and 6 are shared by the two patient groups).

	FVC	Onset Site	ALT	Chloride	Severe Patients (%)	Mild Patients (%)
1	low	bulbar	high	normal	16.04	1.40
2	low	bulbar	high	low	12.74	0.14
3	low	limb	high	normal	10.38	8.64
4	moderate–high	bulbar	high	normal	6.60	1.68
5	low	limb	high	low	6.13	2.38
6	moderate–low	limb	high	normal	6.13	11.67
7	moderate–high	limb	high	normal	2.83	12.51
8	high	limb	high	normal	0.47	12.14
9	moderate–low	limb	normal	normal	0.94	4.72
10	moderate–high	limb	normal	normal	0.00	4.44
